# The effect of negative emotions on smoking craving: the chain-mediating role of self-control and self-exempting beliefs

**DOI:** 10.3389/fpsyt.2025.1642341

**Published:** 2025-09-02

**Authors:** Yixin Zhao, Yong Li, Yang Shu, Xiaoyan Wang, Wen Wang, Lian Yang

**Affiliations:** School of Public Health, Chengdu University of Traditional Chinese Medicine, Chengdu, China

**Keywords:** negative emotions, smoking craving, self-control, self-exempting beliefs, current smokers

## Abstract

**Background:**

Previous studies have demonstrated that negative emotions increase smoking cravings. To date, the specific action pathways between negative emotions and smoking cravings remain unclear. This study aimed to investigate the association between negative emotions and smoking cravings, as well as to determine whether self-control and self-exempting beliefs serve as mediating variables for this association.

**Methods:**

A cross-sectional study was conducted in Sichuan Province, Southwest China, from January 2022 to April 2023. Negative emotions, self-control, self-exempting beliefs, and smoking cravings were assessed using questionnaires. Descriptive statistics and one-way analysis of variance were used to analyze smoking cravings and their influencing factors. Correlation and mediation effect analyses were conducted to investigate the intrinsic correlations among the research variables.

**Results:**

This study involved 1293 current smokers. We observed significant differences in smoking cravings across demographic groups. In addition, negative emotions, self-control, and self-exempting beliefs were all significantly associated with smoking cravings (p< 0.05). After controlling for socio-demographic variables, both self-control and self-exempting beliefs exhibited a chain mediating role between negative emotions and smoking cravings. Negative emotions exerted a direct effect on smoking cravings, with an effect value of 0.0694, accounting for 61.29% of the total effect value. Self-control and self-exempting beliefs mediated the relationship between negative emotions and smoking cravings, with effect values of 0.0334 and 0.0081, representing 29.47% and 7.16% of the total effect value, respectively. The chain mediating effect value of self-control and self-exempting beliefs was 0.0024, representing 2.08% of the total effect value.

**Conclusion:**

This study demonstrates that self-control and self-exempting beliefs partially mediate the relationship between smokers’ negative emotional states and their cravings to smoke. It is important to pay timely attention to the emotional changes of smokers, enhance their ability to maintain self-control in negative emotional states, and mitigate smoking-related self-exempting beliefs to help smokers better cope with smoking cravings caused by emotional changes, prevent relapse, and achieve long-term smoking cessation goals.

## 1 Introduction

Smoking is one of the most critical and preventable risk factors contributing to chronic noncommunicable diseases and premature death worldwide (1). It is estimated that more than 8 million people die annually from smoking-related illnesses, and if current trends persist, this could result in over 1 billion deaths in the 21st century (2–4). The life-threatening hazards and economic losses caused by smoking mainly occur in low- and middle-income countries, including China. Statistics show that Chinese people consume 40% of the world’s tobacco and 26.6% of people aged 15 and above in China are smokers (50.5% of males and 2.1% of females), accounting for approximately one-third of the world’s total smoking population (5–7). Smoking is a major risk factor for a range of serious health conditions, including cancer, heart disease, stroke, lung disease, and chronic obstructive pulmonary disease. However, as of 2018, only 23.2% of adult smokers in China had attempted to quit smoking within the past 12 months (8, 9). A nationwide smoking cessation cohort study in China reported that 21.74% of smokers successfully quit smoking at 24 weeks after receiving professional smoking cessation treatment, whereas the long-term smoking cessation success rate in the general population was less than 3% (10). The reasons for smoking cessation failure mainly include negative affect (e.g., anxiety) and craving (11). Craving, a strong impulse or urgent desire of an individual to use a substance, is recognized as one of the core diagnostic criteria for substance use disorders (DSM-5) (12–14). In the current conceptualization of addiction, craving is considered to be the main driving force behind the continued use of addictive substances such as drugs and cigarettes, as well as relapse after substance abstinence. Among smokers, craving for cigarettes has been identified as a major impediment to smoking cessation and is known to persist long after successful cessation (15–17). Therefore, An in-depth investigation into the factors influencing the craving for smoking is of vital public health significance, as it can inform the development of scientifically formulated smoking cessation intervention strategies, promoting the achievement of long-term smoking cessation goals, and effectively preventing smoking relapse.

### 1.1 The relationship between negative emotions and smoking craving

Negative emotions, such as anxiety, depression, and stress, are among the most common emotional problems and psychological disorders worldwide, and their related impact has become increasingly prominent in recent years (18). Current research indicates that negative emotions are recognized as direct factors associated with smoking cravings, such as health-related anxiety (e.g., COVID-19-related anxiety) may exacerbate smoking urges, particularly under the moderating effects of demographic variables such as age and income level (19, 20). Several studies have demonstrated that relieving emotional distress—particularly feelings of depression and anxiety—is the primary motivation for smoking among some smokers (21, 22). The negative reinforcement theory of drug addiction suggests that addicts tend to resort to substance use to escape or avoid negative emotional states, such as withdrawal symptoms or stress, which may lead to the craving for substance use (23). In addition, some smokers considered mood dysregulation due to negative emotions as a major trigger for smoking behavior, and studies have shown that negative emotions are associated with intensified smoking cravings, especially during periods of smoking deprivation (24). Although multiple studies have demonstrated the association between negative emotions and smoking cravings, the specific pathways of this association remain to be investigated (25–27). Therefore, we proposed the following hypothesis:

Hypothesis 1: Negative emotions positively influence the smoking craving among smokers.

### 1.2 The mediating role of self-control

To date, the specific action pathways linking negative emotions to smoking cravings remain controversial (28). Negative emotions have been clearly shown to impair self-control and are recognized as major triggers of self-control failure (29). Studies have revealed that successful self-control acts as a key protective factor against addictive behaviors, whereas failures in self-control are associated with increased cravings for smoking (30). Among smokers, unfavorable factors such as withdrawal symptoms, strong cravings, as well as environmental and emotional triggers hinder long-term smoking cessation (31). According to the strength model of self-control, the constant effort required to cope with smoking cessation barriers (e.g. cravings and emotional changes) during long-term smoking cessation may deplete self-control strength. This depletion can hinder smokers from maintaining a sustained level of self-control, ultimately increasing the likelihood of relapse This is reflected by the fact that most smokers relapse within one week after quitting smoking (31–33). A study on self-control depletion demonstrated that participants with reduced self-control levels exhibited significantly increased relapse rates, as well as higher smoking cravings during smoking cessation attempts (34). Furthermore, negative emotions such as anxiety, stress, and depression accelerate the depletion of self-regulatory resources. Severe negative emotions may further impair individuals’ ability to suppress emotional impulses, resulting in a complete depletion of self-control, which ultimately may manifest as heightened smoking cravings (35–37). Therefore, the following research hypothesis was proposed:

Hypothesis 2: Self-control is a mediator between negative emotions and smoking craving.

### 1.3 The mediating role of self-exempting beliefs

Like self-control, self-exempting beliefs (SEBs) are a key factor affecting smoking cravings. SEBs are a type of rationalized belief that individuals develop to reduce cognitive dissonance caused by inconsistency between cognition and behavior (38). In smokers, SEBs focus on the notion that they can be exempted from the health risks of smoking (39–41). Cognitive dissonance is the premise for the formation of SEBs. When confronted with the conflict between the correct cognition that smoking is harmful to health and their continued smoking behavior, smokers often resolve the dissonance by modifying their correct cognition rather than the more difficult smoking behavior. Studies have shown that most smokers believe that smoking is beneficial—for example, that it helps regulate negative emotions and improve social interaction (42–44). Self-exempt beliefs influence decision-making by reducing smokers’ experiences of cognitive dissonance and anxiety, thereby hindering smoking cessation. Specifically, smokers with negative emotions are more likely to develop smoking self-exempt beliefs to alleviate emotional disorder, which may subsequently intensify smoking cravings (45). Therefore, the following research hypothesis was proposed:

Hypothesis 3: Self-exempting belief is a mediator between negative emotions and smoking craving.

### 1.4 Chain mediating effects of self-control and self-exempting beliefs

The effects of self-control and self-exempting beliefs on smoking do not exist independently but may interact to form a chain mediation pathway linking negative emotions and smoking cravings. The dual-process models of addiction propose that addictive behaviors stem from an imbalance between the impulsive system (automatic and emotion-driven) and the reflective system (controlled and goal-oriented) (46). The impulsive system drives rapid responses to reward-related cues, such as cravings, while the reflective system regulates behavior through self-control (47). When negative emotions weaken the reflective system’s self-control capacity, the impulsive system dominates, triggering cravings and relapse. Additionally, research shows that individuals with low levels of self-control are more likely to develop irrational cognition, leading to addictive behaviors (48). Self-control failures may lead smokers to develop self-exempting beliefs (e.g., “occasional use is harmless”) to rationalize cravings caused by the impulsive system’s excessive activation (49). Therefore, the following research hypothesis was proposed:

Hypothesis 4: self-control and self-exempting beliefs form a chain mediation pathway between negative emotions and smoking craving among smokers.

**Figure 1:**
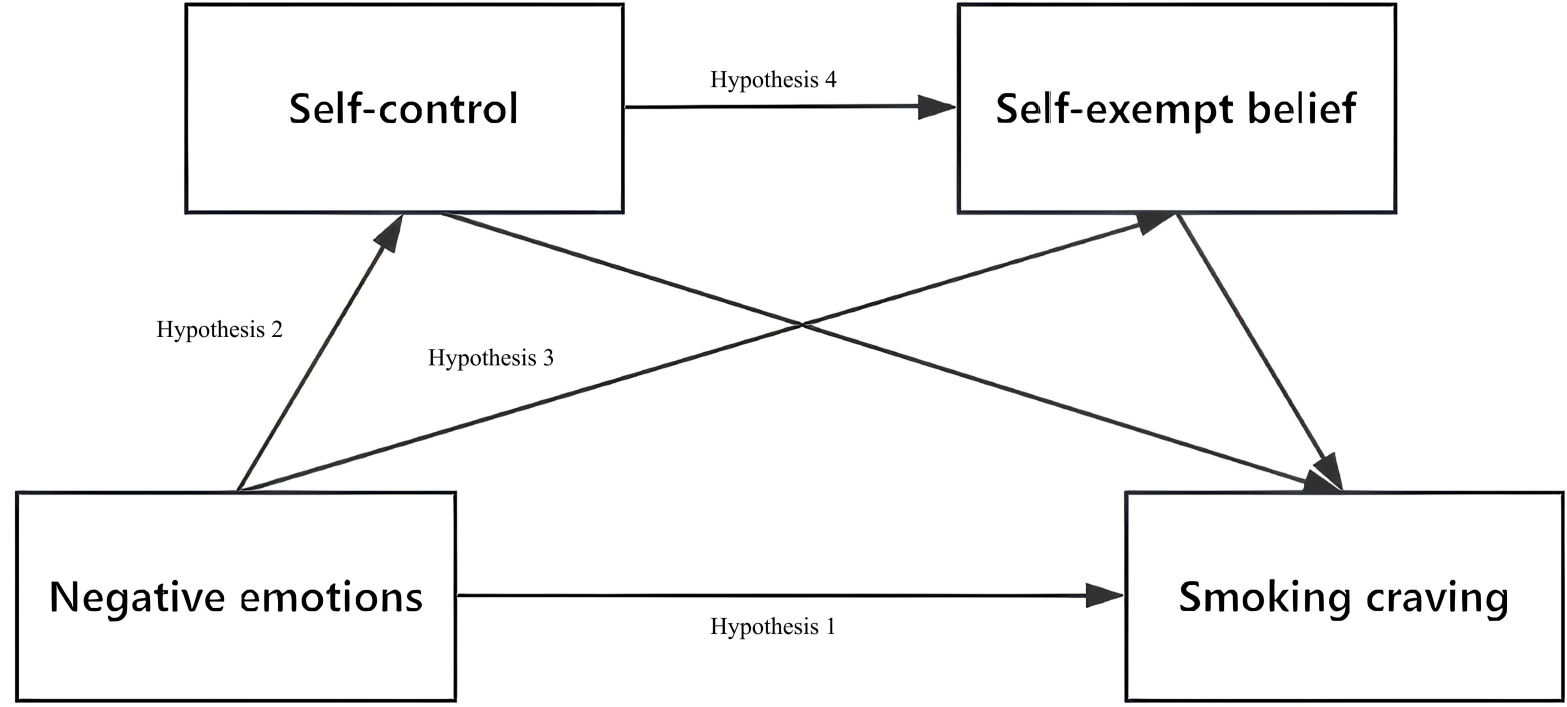
Hypothesized chain mediation model. Hypothesis 1: negative emotions→smoking craving; Hypothesis 2: negative emotions→self-control→smoking craving; Hypothesis 3: negative emotions→self-exempting beliefs→smoking craving; Hypothesis 4: negative emotions→self-control→self-exempting beliefs→smoking craving.

In summary, this study attempts to examine the relationship between negative emotions and smoking craving and to construct a chain mediation model (see **Figure 1**). This study will provide reference information for policymakers and support the timely initiation of intervention strategies under permissible conditions, to better assist smokers in coping with difficulties such as cravings and emotional changes in smoking cessation environments, prevent relapse promptly, and promote the achievement of long-term smoking cessation goals.

## 2 Methods

### 2.1 Participants and procedure

This study was a cross-sectional household-based survey conducted in Sichuan Province, China from January 2022 to April 2023. A stratified multi-stage sampling strategy was adopted in the survey. Based on the province’s geographical distribution, economic development, and health indicators, four representative prefecture-level cities or autonomous prefectures were selected as the survey areas: Wenjiang District of Chengdu City, Fushun County of Zigong City, Qingchuan County of Guangyuan City, and Xide County of Liangshan Prefecture. For sampling purposes, two streets and two townships were selected from each of the four areas, with two communities and two villages from each street and township, respectively.

This survey encompassed an entire population sample of 6189 individuals, including non-smokers. The study inclusion and exclusion criteria are as follows: (1) Aged 15 years or older; (2) Exclusion of female participants who are pregnant or currently breastfeeding; (3) Absence of psychiatric comorbidities. To minimize self-reporting bias, current smokers were defined using both subjective and objective criteria: (1) self-reported lifetime cigarette consumption ≥100 cigarettes and smoking within the past 30 days, and (2) salivary cotinine levels >10 ng/mL (50). Among the 6189 respondents, 1410 (22.78%) identified as current smokers. To avoid estimation bias, questionnaires with missing values were considered invalid and excluded during quality control. Finally, the valid sample included 1293 current smokers, yielding an overall validity rate of 91.70% (1293/1410). The survey was conducted face-to-face. All participants completed a questionnaire that included basic demographic information (e.g., age, gender, marital status, education level, and household registration), the Self-Control Scale (SCS), the Depression-Anxiety-Stress Self-Rating Scales (DASS-21), and the SEB scale. Each survey session was conducted by fully trained investigators to ensure quality control. All subjects voluntarily participated in this study and provided informed consent. The study protocol was approved by the Ethics Committee of Chengdu University of Traditional Chinese Medicine (Approval No.: 2023KL-134).

### 2.2 Measures

#### 2.2.1 Sociodemographic characteristics

This survey collected basic demographic data using a questionnaire, which included the following variables: participants’ gender (male/female), age (15~25~35~45~55~65), alcohol consumption (yes/no), education level (illiterate/primary school/secondary school/high school/university or above), household registration (urban/rural), employment status (employed/retired/in school/unemployed), and ethnicity (Han Chinese/other).

#### 2.2.2 Negative emotions

Negative emotion levels were measured using the simplified Chinese version of the DASS-21 (51, 52). The DASS-21 consists of 21 items divided equally into three subscales—measuring depression, anxiety, and stress factors—each containing 7 items. The items were scored on a 4-point response scale ranging from 0 to 3 (0 = never, 1 = sometimes, 2 = often, 3 = always), with higher scores indicating stronger emotional experience. This scale has been previously used to quantitatively assess negative emotion levels of subjects (53) and demonstrated good internal consistency in this study (Cronbach’s α = 0.909).

#### 2.2.3 Self-control

Self-control was measured using the Chinese version of the SCS. Originally published in 2004, the SCS was later revised by Chinese scholars and has since been widely used to assess self-control in Chinese populations (54). The scale consists of 19 items and covers five dimensions involving impulse control, healthy habits, resistance to temptation, focus on work, and moderate entertainment. The scale employs a five-point Likert scoring system, ranging from 1 (completely disagree) to 5 (completely agree), and includes 15 reverse scoring questions. The total score ranges from 19 to 95, with higher scores indicating greater self-control. In this study, after reverse scoring all reverse-scored items in the questionnaire, the Cronbach’s alpha coefficient of the questionnaire was 0.824, indicating good reliability.

#### 2.2.4 Self-exemption beliefs

The simplified Chinese version of the SEB scale was used to assess the participants’ SEBs. The scale consists of 10 items, including statements such as “Smoking is nothing nowadays as everything can cause cancer “, “Life is short and smoking brings more pleasure than harm “, and “Many things are predetermined, and smoking is not a big deal” (55, 56). Each item was scored on a 5-point Likert scale ranging from 1 (strongly disagree) to 5 (strongly agree). In this study, the scale demonstrated good internal consistency (Cronbach’s α = 0.931).

#### 2.2.5 Smoking craving

Smoking craving refers to the strong impulse or desire of smokers to use cigarettes. Craving intensity was assessed using a single visual sliding scale based on the answer of the research subjects to the following question: “If 0 is very weak and 10 is very strong, how would you rate your smoking craving over a 24-hour period?”. The scale ranged from 0 on the left side to 10 on the right side, with higher scores indicating stronger cravings. Our study was conducted as a large-scale, household-based epidemiological survey across multiple counties in Sichuan Province. In this context, the VAS provided a concise and efficient tool for measuring craving while minimizing respondent burden. Given the comprehensive nature of the questionnaire (which included demographic, emotional, cognitive, and behavioral variables), brevity was essential to maintain participant engagement and reduce fatigue. A similar question to that in this study has been previously used to quantify the extent of smoking cravings among smokers, and it has demonstrated strong correlations with multi-item scales such as the QSU (57, 58).

**Table 1** presents the scale properties of each dimension for the study variables.

**Table 1:** Definition of each variable.

Variable types and variable names	Number of Items	Variable definition	Cronbach’s α
Explanatory variable			
Negative emotions (DASS - 21)	21	Negative emotions score ranged from 0 to 126	0.909
Anxiety	7	Anxiety score ranged from 0 to 42	0.806
Depression	7	Depression score ranged from 0 to 42	0.881
Stress	7	Stress score ranged from 0 to 42	0.834
Explained variable			
Smoking craving	1	Smoking craving score ranged from 0 to 10	
Mediator variables			
Self-control (SCS)	19	Self-control score ranged from 19 to 95	0.824
Impulse control	6	Impulse control score ranged from 6 to 30	0.758
Healthy habits	3	Healthy habits score ranged from 3 to 15	0.651
Resistance to temptation	4	Resistance to temptation score ranged from 4 to 20	0.636
Focus on work	3	Focus on work score ranged from 3 to 15	0.527
Moderate entertainment	3	Moderate entertainment score ranged from 3 to 15	0.609
Self-exemption beliefs (SEBs)	10	Self-exemption beliefs score ranged from 10 to 50	0.931

### 2.3 Statistical analysis

Data analysis was carried out using SPSS version 26.0. First, descriptive statistics were calculated for sociodemographic variables. Second, Mann–Whitney U and Kruskal–Wallis H were then used to assess the differences in smoking cravings across the various respondent subgroups. Subsequently, the data were standardized, and Spearman correlation analysis was conducted to examine the correlation between negative emotions, self-control, SEBs, and smoking cravings. Finally, the mediating effects of self-control and SEBs among negative emotions and smoking cravings were analyzed using Model 6 of the PROCESS macro developed by Hayes, with gender, age, education level, drinking habits, household registration, ethnicity, and employment status incorporated into Model 6 as control variables (59). The Bootstrapping method with 5000 repeated samplings was used to generate the 95% confidence interval for the parameter estimates, with intervals excluding 0 indicating a statistically significant mediation effect (p<0.05).

## 3 Results

### 3.1 Common method bias test

Common method bias was tested using Harman’s one-way test (60), because the data were self-reported by the participants. The analysis revealed 10 common factors with eigenvalues greater than 1, extracted without rotation. The first factor accounted for 18.42% of the variance, significantly below the 40% threshold, suggesting that serious common method bias was not present.

### 3.2 Characteristics of samples

As shown in **Table 2**, the average smoking craving index among the respondents was 6.26 ± 2.11, with males exhibiting significantly greater craving for smoking than females (p<0.05). Craving levels also differed significantly across groups of age, education levels, employment status, and household registration types (p<0.05). Specifically, middle-aged and older adults (≥45 years) reported significantly higher cravings for cigarettes than young smokers (p<0.001). Education level was inversely correlated with smoking cravings, with individuals possessing lower education attainment reporting stronger smoking cravings (p<0.001). Smokers who had been in the workforce, regardless of whether they were currently employed or temporarily unemployed, exhibited a stronger craving for smoking than students (p<0.001). Rural smokers reported higher cravings than their urban counterparts (p<0.05). Notably, no statistical correlation was identified between alcohol consumption and smoking cravings. Based on these findings, we included gender, age, education level, drinking habits, household registration, ethnicity, and employment status as control variables in the mediation analysis using the PROCESS macro. Although no direct association was observed between drinking and smoking cravings, its inclusion was retained due to the high prevalence in the population and evidence from previous studies (61).

**Table 2:** Association between demographic variables and smoking craving: a univariate analysis of cross-sectional survey data.

Variables	Category (%)	Smoking desire ( $\bar{x}\pm s$ )	Z/H	P	Paired comparison
Age					
	15~(13.9)	5.58 ± 2.21	51.352^a^	<0.001	④,⑤,⑥>① ④,⑤,⑥>②
	25~(15.4)	5.81 ± 1.97			
	35~(12.0)	6.15 ± 2.16			
	45~(19.9)	6.51 ± 1.99			
	55~(16.1)	6.64 ± 2.09			
	65~(22.7)	6.56 ± 2.08			
Gender					
	Male(94.0)	6.30 ± 2.08	-2.181^b^	<0.05	
	Female(6.0)	5.69 ± 2.48			
Education level					
	Illiterate(9.0)	6.66 ± 2.12	31.834^a^	<0.001	①>④ ②>④
	Primary & Secondary Education(57.8)	6.41 ± 2.05			
	High School/Vocational School(17.3)	6.13 ± 2.21			
	College or Higher(15.9)	5.63 ± 2.05			
Occupational Status					
	In the Workforce(63.3)	6.21 ± 2.05	22.723^a^	<0.001	①>③ ②>③ ⑤>③
	Retired(10.4)	6.74 ± 1.98			
	Student(7.0)	5.50 ± 2.21			
	Unemployed(0.8)	6.50 ± 1.90			
	Not in Labor Force(18.5)	6.46 ± 2.25			
Residence Type					
	Rural(71.2)	6.34 ± 2.09	-2.186^b^	<0.05	
	Urban(28.8)	6.07 ± 2.13			
Alcohol Use Status					
	Current Drinker(44.8)	6.36 ± 2.04	-1.529^b^	0.131	
	Non-drinker(55.2)	6.15 ± 2.18			
Ethnicity					
	Han Ethnicity(85.6)	6.15 ± 2.18	-2.048^b^	<0.05	
	Other Ethnicities(14.4)	6.22 ± 2.13			
Total		6.26 ± 2.11			

^a^Kruskal-Wallis test. ^b^Mann-Whitney test.①-⑥ mean different groups.

### 3.3 Correlation of negative emotions, self-control, self-exempting beliefs, and smoking craving

**Table 3** presents the results of correlation analyses between negative emotions, self-control, SEBs, and smoking cravings. Specifically, negative emotions exhibited a significant negative correlation with self-control (r=–0.341, p<0.01) and a significant positive correlation with SEBs (r=0.072, p<0.01) and smoking cravings (r=0.062, p<0.05). In addition, self-control was negatively correlated with both SEBs (r=–0.096, p<0.01) and smoking cravings (r=–0.112, p<0.01), whereas SEBs were positively correlated with smoking cravings (r=0.128, p<0.01). Overall, this study revealed significant correlations among all variables, providing a basis for subsequent mediation effect tests.

**Table 3:** Correlation analysis between negative emotions, self-control, self-exempting beliefs and smoking craving.

Variable	Negative emotions	Self-control	Self-exempting beliefs	Smoking craving
negative emotions	1.000			
self-control	-0.341^**^	1.000		
self-exempting beliefs	0.072^**^	-0.096^**^	1.000	
smoking craving	0.062^*^	-0.112^**^	0.128^**^	1.000

**Correlation is significantat the 0.01 level(2-tailed). *Correlation is significantat the 0.05 level(2-tailed).

### 3.4 Chain mediation model test

Model 6 in the Process macro program developed by was utilized to examine the chain mediation model (see **Table 4**). The results indicated that negative emotions significantly predicted smoking craving (β=0.11, p<0.001). When self-control and self-exempting beliefs were introduced as mediators, negative emotions remained significant in predicting smoking craving (β=0.07, p<0.01); negative emotions significantly predicted self-control (β=−0.30, p<0.001) and self-exempting beliefs (β=0.10, p<0.01); and self-control significantly predicted self-exempting beliefs (β=−0.10, p<0.01). Furthermore, both self-control (β=−0.11, p<0.01) and self-exempting beliefs (β=0.08, p<0.01) reached significant levels of predictive power for smoking craving.

**Table 4:** Results of regression analysis.

Regression equation		Overall fit indices			Significance of the regression coefficients		
Outcome variables	Predictors	R	R²	F	β	95%CI	t
Smoking cravings	Negative emotions	0.24	0.06	9.99***	0.11	[0.06,0.17]	4.08***
Self-control	Negative emotions	0.35	0.12	22.16***	-0.30	[-0.35,-0.25]	-11.27***
Self-exempting beliefs	Negative emotions	0.23	0.05	7.62***	0.10	[0.04,0.16]	3.48**
	Self-control				-0.10	[-0.16,-0.04]	-3.38**
Smoking cravings	Negative emotions	0.28	0.08	10.69***	0.07	[0.01,0.13]	2.40**
	Self-control				-0.11	[-0.17,-0.05]	-3.84**
	Self-exempting beliefs				0.08	[0.03,0.13]	2.90**

**p<0.01, ***p<0.001. Gender, age, home location, alcohol consumption, education level, household registration, employment status were done as control variables incduded in the model. The study varables were standardized.

### 3.5 Mediation analysis of self-control and self-exempting beliefs

**Figure 2:**
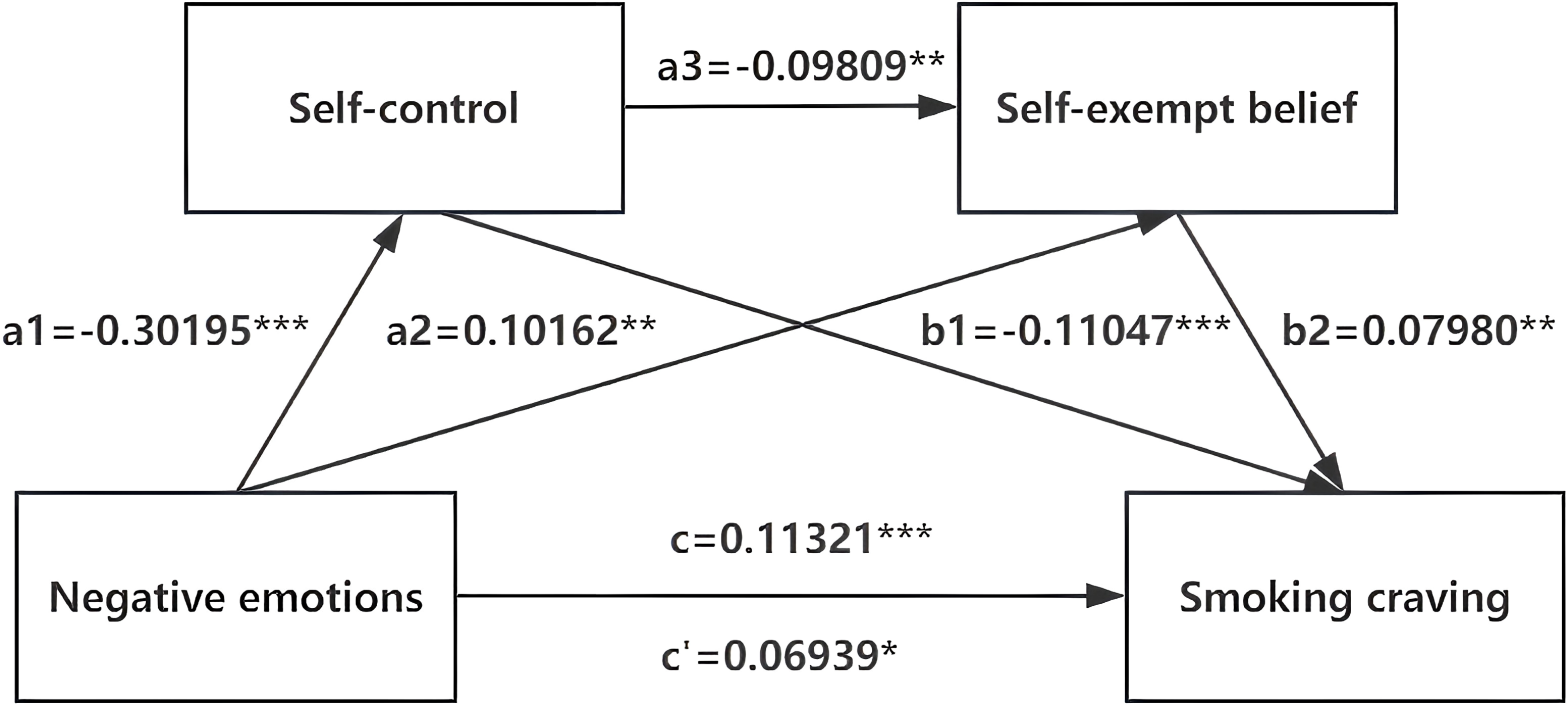
The chain mediating effect of self-control and self-exempting beliefs on the negative emotions and smoking craving. *p<0.05,**p<0.01,***p<0.001.

The above analyses revealed significant correlations between negative emotions, self-control, SEBs, and smoking cravings, prompting us to investigate the mediating effect involving these variables. After controlling for demographic characteristics, the chain mediation analysis revealed that the negative emotions of smokers had a significant effect on their smoking cravings (p<0.001), with an overall effect value of 0.113 (see **Figure 2**, **Table 5**). This finding suggests that smokers experienced an increased craving for smoking when facing high levels of negative emotions such as anxiety, depression, and stress. In addition, all direct and indirect pathways yielded significant results. Among all effect values, the direct effect value was 0.069, accounting for 61.29% of the total effect value, and the total indirect effect value was 0.044, accounting for 38.71% of the total effect value. Specifically, the first indirect pathway revealed a significant mediating effect of self-control on the relationship between negative emotions and smoking cravings, with an effect value of 0.033, accounting for 29.47% of the total effect value. The second pathway showed that SEBs had a significant effect on mediating the relationship between negative emotions and smoking cravings, with an effect value of 0.008, accounting for 7.16% of the total effect value. The third pathway demonstrated a chain mediation effect of self-control and SEBs between negative emotions and smoking craving, with an effect value of 0.002, accounting for 2.08% of the total effect value. A significant negative correlation was observed between self-control and SEBs, with an effect value of 0.098. Altogether, these data suggest that self-control and SEBs partially mediate the relationship between negative emotions and smoking cravings, exerting a chain mediation effect.

**Table 5:** The chain mediating model of self-control and self-exempting beliefs between negative emotions and smoking craving.

Pathway	Effect	SE	BootLLCI	BootULCI	Relative mediation effect
Total effect(c)	0.11321	0.02773	0.05882	0.16761	
Direct effect(c’)	0.06939	0.02894	0.01261	0.12616	61.29%
a1	-0.30195	0.02679	-0.35450	-0.24939	
a2	0.10162	0.02920	0.04433	0.15890	
a3	-0.09809	0.02902	-0.15502	-0.04116	
b1	-0.11047	0.02875	-0.16687	-0.05406	
b2	0.07980	0.02754	0.02577	0.13383	
Indirect effects					
Total indirect effects	0.04383	0.01074	0.02427	0.06638	38.71%
Indirect1	0.03336	0.01000	0.01510	0.05450	29.47%
Indirect2	0.00811	0.00373	0.00183	0.01638	7.16%
Indirect3	0.00236	0.00117	0.00047	0.00504	2.08%

Indirect1: negative emotions→self-control→smoking craving; Indirect2: negative emotions→self-exempting beliefs→smoking craving; Indirect3: negative emotions→self-control→self-exempting beliefs→smoking craving. BootLLCI, bootstrapping lower limit confidence interval; BootULCI, bootstrapping upper limit confidence interval; SE, standard error; Effect, standardized regression coefficient.

## 4 Discussion

In this study, we demonstrated that negative emotions had a significant effect on current smokers’ craving for smoking with higher levels of negative emotions associated with stronger cravings. Moreover, self-control and Self-exempting beliefs exerted a significant chain mediating effect in the relationship between negative emotions and smoking cravings. All these findings support our research hypotheses.

### 4.1 The direct effect of negative emotions on smoking craving

This study established that negative emotions significantly predicted smoking cravings. This finding is consistent with those of previous studies (26, 62, 63). The effect of negative emotions on smoking cravings warrants further investigation through both theoretical and clinical neuroscience studies. Theoretically, craving is believed to be the basis of addictive behavior (64). Within the research field on addictive motivation, negative reinforcement theory is one of the earliest studies and is defined as “the desire to escape or avoid negative emotions” (23). In this case, addicts wish to alleviate negative emotions through the use of drugs or cigarettes, which may lead to cravings. In addition, clinical neuroscience laboratory evidence suggests that nicotine can activate nicotinic acetylcholine receptors (nAChRs) and increase the release of various neurotransmitters such as dopamine, serotonin, and GABA in the body, thus creating feelings of excitement and pleasure, while simultaneously reducing anxiety and tension (65). Hence, individuals may use smoking to cope with negative emotions or may smoke in anticipation that it will help them manage emotionally distressing events. In the future, more attention should be given to the role of regulating negative emotions in reducing smoking and supporting smoking cessation efforts.

### 4.2 The mediation effect of self-control and self-exempting beliefs

The results also suggest that self-control plays a mediating role in the relationship between negative emotions and smoking cravings. In other words, negative emotions can not only directly affect smokers’ smoking cravings, but they can also influence this behavior through depletion of self-control. These observations are consistent with the performance of the strength model of self-control. Smokers experiencing higher levels of negative emotions tended to exhibit reduced self-control, which in turn elevates the intensity of their cravings, leading to continued smoking behavior and difficulties in quitting. Previous research has shown that smokers’ cravings for cigarettes are associated with the strength of self-control and that the depletion of self-control leads to an increase in smoking attempts. Over time, self-control may weaken due to stress, strong cravings for smoking, and the extra effort required to manage negative emotions through means other than smoking. This cumulative strain can result in self-control fatigue and reduced ability to suppress the craving for smoking. Therefore, the accumulation of negative emotions, such as stress, may predict the depletion of self-control, which creates cravings for smoking (32, 66). A clinical trial demonstrated that smoking cravings—due to decreased self-control—increased during the first two weeks of a quitting attempt and were positively correlated with negative emotions (67). A neuroscience study was conducted to examine how negative emotions weaken self-control. Concretely speaking, it revealed that individuals with negative urgency traits exhibit overactivation in brain regions associated with inhibitory control (e.g., prefrontal cortex) when experiencing negative emotions. This overactivation may be a compensatory response. However, excessive neural activity may lead to long-term depletion of self-regulatory resources, which in turn predicts subsequent self-control failures, such as substance abuse. These findings suggest that excessive brain regulatory resource responses may contribute to individuals frequently experiencing self-control failures due to negative emotions (35).

Likewise, this study validated that negative emotions can predict smoking cravings through the mediating role of self-exempting beliefs. This result can be explained through the theory of cognitive dissonance. Cognitive dissonance is the premise for the emergence of SEBs. Smokers may experience psychological discomfort and stress when their behavior (smoking) contradicts their cognition (smoking is harmful). SEBs function as rationalizations that reduce this dissonance by altering beliefs (e.g., “I smoke, but it helps me manage stress, so it’s not that bad) (39). Research shows that smokers experience cognitive dissonance during negative emotions as the direct conflict between their behavior and health-related cognitions is amplified by emotion. While smoking is frequently used to regulation emotion in these situations, it creates a contradiction when smokers confront the cognition of smoking-related health harm (68). Moral disengagement theory helps explain how smokers selectively override moral standards. Self-exempting beliefs act as psychological tools that allow continued smoking without guilt—either by shifting blame (“Male-specific social contexts force me to smoke”) or downplaying risks (“A few cigarettes won’t hurt”). To resolve this mental conflict, smokers might highlight their struggles with quitting-related stress, using this justification to produce cravings (69).

### 4.3 The serial multiple mediation models

Moreover, the results showed that negative emotions affect self-control, and self-control failure triggers self-exempting beliefs, which, in turn, contributes to smoking cravings. Negative emotions deplete self-control, further prompting individuals to develop rationalizing beliefs. According to the dual-process model of addiction, addictive behaviors are influenced by the impulsive system and the reflective system. When faced with negative emotions, smokers are more likely to activate the impulsive system and exhibit wrong decision-making (70). Additionally, negative emotions deplete self-control resources, thereby inhibiting the reflective system. When self-control resources are insufficient, individuals typically generate self-exempting beliefs, a cognitive bias rationalizing smoking behavior. Thus, negative emotions not only accelerate the depletion of smokers’ self-control capacity but also amplify cognitive dissonance, thereby generating and maintaining smoking cravings (71). Notably, although the chain-mediated effect appears modest in magnitude, it offers meaningful theoretical and practical insights. Specifically, this sequential pathway highlights how negative emotions activate smoking cravings through cognitive resource depletion (i.e., diminished self-control capacity) and cognitive dissonance (i.e., generation of self-exempting beliefs). This dual-path explanation integrates cognitive and resource-depletion mechanisms, enriching existing literature and providing a more comprehensive perspective on how negative emotions influence addictive symptoms.

### 4.4 Limitations and perspectives

The findings of this study contribute to a better understanding of the psychological mechanisms underlying the association between negative emotions and smoking cravings among current smokers. From a practical perspective, these findings may inform the development of smoking cessation strategies by guiding the design of psychologically grounded interventions or smoking cessation policies. Specifically, this study highlights potential smoking cessation countermeasures (e.g., timely regulation of negative emotions, exercise of self-control, and development of correct cognition) to mitigate withdrawal-related cravings and reduce the risk of relapse during smoking cessation (70). There are some limitations to this study. First, this study assessed negative emotions, self-control, SEBs, and smoking cravings through self-reports. Such assessment may result in overestimation or underestimation of the parameters compared to objective measurements (e.g., laboratory tests and detection of biochemical indicators). We used the Visual Analog Scale (VAS) to measure smoking craving. While the VAS is suitable for field use, it does not capture the multi-dimensional structure of craving (e.g., anticipation, relief, intention) like the Questionnaire of Smoking Urges-Brief (QSU-Brief). Future studies should explore this association using multidimensional scales. In addition, we did not distinguish whether various types of negative emotions (including anxiety, depression, and stress) have a promoting effect on smoking cravings within the proposed mediating framework. This issue needs to be addressed by more in-depth follow-up studies. Second, the current study adopted a cross-sectional research design, which does not support the assessment of causal relationships between variables. Therefore, future research may employ longitudinal follow-up surveys, experimental manipulation of SEBs, or ecological momentary assessment (EMA) of craving. to further investigate the time series relationship between variables, providing a basis for the development of more scientific tobacco control intervention programs. Finally, other factors such as social support and time preference may play a critical role in the relationship between negative emotions and smoking cravings. In this context, examining the potential mediating and/or moderating roles of these and other variables in the relationship between negative emotions and smoking cravings is recommended to identify a more comprehensive underlying mechanism.

## 5 Conclusion

The present study demonstrated that negative emotions significantly exacerbate smoking cravings, with self-control and SEBs acting as important mediating factors between negative emotions and smoking cravings among current smokers. Smokers may use cigarettes to cope with their negative emotions; however, this increases their craving and relapse. To break this cycle, this study confirmed the influence of negative emotions on smoking cravings, revealed the intrinsic mechanism between self-control and self-exempting beliefs, and is of positive significance for understanding the causes of tobacco addiction behaviors and interventions for smokers facing emotional pressures. Notably, as intervention approaches such as cognitive behavioral therapy (CBT) or mindfulness-based cognitive therapy (MBCT)—which enhance emotional regulation and reduce self-rationalizing beliefs—are becoming increasingly well-established, this study lays a foundation for practical applications targeting emotion regulation in educational and clinical settings to reduce addictive smoking behaviors (72, 73).

## Data Availability

The datasets presented in this article are not readily available due to patient privacy concerns but can be shared on reasonable request. Requests to access the datasets should be directed to Lian Yang, yyanglian@163.com.
